# Efficacy of the Once-Daily Tacrolimus Formulation LCPT Compared to the Immediate-Release Formulation in Preventing Early Post-Transplant Diabetes in High-Risk Kidney Transplant Patients: A Randomized, Controlled, Open-Label Pilot Study (EUDRACT: 2017-000718-52)

**DOI:** 10.3390/jcm13247802

**Published:** 2024-12-20

**Authors:** Armando Torres, Concepción Rodríguez-Adanero, Constantino Fernández-Rivera, Domingo Marrero-Miranda, Eduardo de Bonis-Redondo, Aurelio P. Rodríguez-Hernández, Lourdes Pérez-Tamajón, Ana González-Rinne, Diego Álvarez-Sosa, Alejandra Álvarez-González, Nuria Sanchez-Dorta, Estefanía Pérez-Carreño, Laura Díaz-Martín, Sergio Luis-Lima, Ana E. Rodríguez-Rodríguez, Antonia María de Vera González, Cristina Romero-Delgado, María Calvo-Rodríguez, Rocío Seijo-Bestilleiro, Consuelo Rodríguez-Jiménez, Manuel Arturo Prieto López, Antonio Manuel Rivero-González, Domingo Hernández-Marrero, Esteban Porrini

**Affiliations:** 1 Nephrology Service, Hospital Universitario de Canarias, 38320 La Laguna, Spain; c.rguez.adanero@gmail.com (C.R.-A.); dmarrero72@hotmail.com (D.M.-M.); ebonis@telefonica.net (E.d.B.-R.); aureliopas@hotmail.com (A.P.R.-H.); mpertam28@gmail.com (L.P.-T.); rinneanag@yahoo.es (A.G.-R.); diegoalvarezsosa@yahoo.es (D.Á.-S.); alejandramag73@gmail.com (A.Á.-G.); nuriasanchezdorta@gmail.com (N.S.-D.); manuloprieto1999@gmail.com (M.A.P.L.); domingohernandez@gmail.com (D.H.-M.); 2 Instituto de Tecnologías Biomédicas (ITB), Universidad de La Laguna, 38206 La Laguna, Spain; anarrguez@gmail.com (A.E.R.-R.); estebanporrini72@hotmail.com (E.P.); 3 Servicio de Nefrología, Complejo Hospitalario Universitario de A Coruña, 15006 La Coruña, Spain; constantino.fernandez.rivera@sergas.es (C.F.-R.); maria.calvo.rodriguez@sergas.es (M.C.-R.); rocio.seijo.bestilleiro@sergas.es (R.S.-B.); 4 Research Unit, Hospital Universitario de Canarias, 38320 La Laguna, Spain; estefaniaperezc@gmail.com (E.P.-C.); lauradiazmart@gmail.com (L.D.-M.); 5 Central Laboratory, Hospital Universitario de Canarias, 38320 La Laguna, Spain; luis.lima.sergio@gmail.com (S.L.-L.); adeverag@gmail.com (A.M.d.V.G.); 6 Pharmacy Service, Hospital Universitario de Canarias, 38320 La Laguna, Spain; cromdels@gobiernodecanarias.org; 7 Pharmacology Service, Hospital Universitario de Canarias, 38320 La Laguna, Spain; conrodjim@gmail.com; 8 Nephrology Service, Hospital Universitario Nuestra Señora de la Candelaria, 38010 Tenerife, Spain; arivero61@hotmail.com

**Keywords:** kidney transplantation, post-transplant diabetes, post-transplant prediabetes, tacrolimus pharmacokinetics, early-release tacrolimus, LCPT tacrolimus

## Abstract

**Background/Objectives**: Post-transplant diabetes mellitus (PTDM) and prediabetes (PreDM) are common after renal transplantation and increase the risk of cardiovascular events and mortality. Compared to immediate-release tacrolimus (IR-Tac), the LCPT formulation, with delayed absorption, offers higher bioavailability and a smoother time–concentration curve, potentially reducing beta-cell stress. **Methods**: This randomized pilot trial compared de novo immunosuppression with IR-Tac (twice daily) and LCPT (once daily). At-risk recipients (age ≥ 60 years or 18–59 years with metabolic syndrome) were enrolled and followed for 3 months. The primary and secondary outcomes were the incidence of PTDM and PreDM, respectively. **Results**: 27 patients were randomized to IR-Tac and 25 to LCPT. The incidence of PTDM was comparable between groups [IR Tac: 18.5% (95% CI: 8.2–36.7%) vs. LCPT: 24% (95% CI: 11.5–43.4%); *p* = 0.7]. Although not statistically significant, the LCPT group exhibited a trend toward a reduction in PreDM incidence [IR-Tac: 40.7% (95% CI: 25–59%) vs. LCPT: 20% (95% CI: 9–39%); *p* = 0.1]. A sensitivity analysis showed similar results, with no significant differences in cumulative corticosteroid doses or baseline body mass index (BMI) between groups. The LCPT group showed a trend toward higher tacrolimus exposure at the end of the study [trough levels: IR-Tac group 8.3 (6.9–9.2) vs. LCPT group 9.4 (7.4–11.4) ng/mL; *p* = 0.05)], as well as fewer acute rejection episodes (none vs. three). Delayed graft function was more common in the IR-Tac group (37% vs. 8%; *p* = 0.01), and the eGFR was lower. Adverse events were comparable between groups. **Conclusions**: The potential biological activity of LCPT in preventing glucose metabolic alterations in at-risk patients warrants further investigation.

## 1. Introduction

Post-transplant diabetes mellitus (PTDM) is a serious condition affecting 10–30% of renal transplant recipients [1,2,3]. PTDM is linked to an increased risk of cardiovascular disease [4,5] and decreased patient survival [5]. Additionally, prediabetic states (PreDM: impaired glucose tolerance and/or impaired fasting glucose) are prevalent, with 40–50% of recipients developing PTDM or PreDM [2]. Recipients with PreDM also face a heightened risk of fatal or non-fatal cardiovascular events nearly equivalent to those with PTDM [4]. Mechanistically, a combination of insulin resistance and inefficient pancreatic beta-cell function predisposes individuals to PreDM and PTDM, with immunosuppression exacerbating pre-existing damage [3,6,7]. Tacrolimus, used in both animal models and human islets, exacerbates cellular damage caused by insulin resistance and obesity [8,9,10].

Different formulations of tacrolimus exhibit distinct pharmacokinetics. Immediate-release tacrolimus (IR-Tac), which is administered twice daily, is rapidly absorbed in the proximal small bowel, reaching peak blood concentrations 90 to 120 min post-administration. The once-daily extended-release tacrolimus (ER-Tac, Advagraf^®^, Astellas Pharma, Madrid, Spain) slows the diffusion rate of tacrolimus. Conversely, the once-daily LCPT tacrolimus, developed using MeltDose technology, results in a more distal distribution in the gut and delayed absorption. Compared to IR-Tac and ER-Tac, LCPT has a lower and delayed peak with comparable minimum concentrations and less peak-to-trough fluctuation [11,12,13,14,15]. LCPT also shows greater bioavailability, requiring 30% less dosing than IR-Tac and 36% less than ER-Tac [12]. However, a clear research gap exists regarding the clinical significance of this ’flatter’ time–concentration curve of LCPT. We hypothesize that it may reduce beta-cell stress and, consequently, the incidence of PTDM and/or PreDM compared to a twice-daily formulation.

The aim of this pilot, randomized, open-label trial was to evaluate whether de novo immunosuppression with LCPT reduces the incidence of PTDM compared to IR-Tac. The incidence of PreDM was assessed as a secondary outcome. Patients at risk of PTDM [6] were included and followed for 3 months post-transplantation, a period associated with maximum beta-cell stress and peak incidence of PTDM [2]. To the best of our knowledge, this is the first randomized controlled study comparing these tacrolimus formulations.

## 2. Material and Methods

### 2.1. Study Design

This study was an investigator-driven, open-label, prospective, randomized phase IV clinical trial with a duration of 3 months. De novo renal transplant (RT) patients with low immunological risk and risk for PTDM were randomized 1:1 into two groups: IR-Tac (Prograf^®,^ Astellas Pharma, Madrid, Spain) or LCPT tacrolimus (Envarsus^®^, Chiesi España, Barcelona, Spain). All participants received induction therapy with either basiliximab or rabbit thymoglobulin. Informed consent was obtained from all patients, and the study was approved by the Ethics and Clinical Research Committee of Hospital Universitario de Canarias. This study was Registered in the European Union Clinical Trials Register on 31 March 2017 (EUDRACT: 2017-000718-52).

### 2.2. Study Population

Two transplant centers participated in this study. The included patients had end-stage renal disease, received their first renal transplant (RT), and met the following “Metabolic Criteria”: (a) recipient age ≥ 60 years or (b) recipient age between 18 and 59 years with metabolic syndrome (defined by the presence of at least three of the following criteria: BMI > 28 kg/m^2^, treated hypertension, pre-transplant fasting glucose levels of 100–125 mg/dL, pre-transplant triglycerides levels ≥ 150 mg/dL, or HDL cholesterol levels < 40 mg/dL in men or <50 mg/dL in women). Exclusion criteria included pre-transplant diabetes (defined as baseline blood glucose level ≥ 126 mg/dL or the use of hypoglycemic medications), dual-organ transplantation, high immunological risk, infection with hepatitis B and/or C viruses, positive pregnancy test, or planned administration of non-standard immunosuppressive regimens (e.g., reduced tacrolimus exposure with mTOR inhibitors).

### 2.3. Randomization, Groups, and Interventions

After signing informed consent, patients were randomized 1:1 using a computerized algorithm generated by the Research Unit of Hospital Universitario de Canarias [16]. Due to the COVID-19 pandemic, the recruitment period was extended, beginning on 30 August 2017 and concluding on 5 April 2022.

- Group 1: IR-Tac: 0.1 mg/kg/day in 2 divided doses to maintain tacrolimus trough levels at between 8–12 ng/mL for the first month. A single dose of 0.05 mg/kg or 0.10 mg/kg (in expanded criteria or standard donor, respectively) was administered before surgery.
- Group 2: LCPT tacrolimus: 0.1 mg/kg/day in a single dose starting within the first 24 h after transplantation to maintain tacrolimus trough levels at between 8–12 ng/mL for the first month.

Doses of IR-Tac and LCPT were adjusted after the first month to maintain tacrolimus levels at between 6–10 ng/mL. Both groups also received additional immunosuppression with:

- Mycophenolate mofetil (MMF) 2 g/day or mycophenolic acid (EC-MFA) 1.44 g/day during the first month, then reduced to 1 g/day or 720 mg/day, respectively.
- Corticosteroids at reduced exposure as previously described, with slight modifications [16]: intravenous methylprednisolone 0.25 g intraoperatively and 60 mg on day 1; oral prednisone starting on day 3 with 20 mg/day, progressively reduced to 5 mg/day from day 42 until the end of the study.
- Induction was based on basiliximab, 20 mg intravenously on days 0 and 4. For grafts at high risk of delayed graft function (e.g., donation after cardiac death or prolonged cold ischemia time), a transient reduction of tacrolimus doses or delayed initiation (maximum of 5 days) plus induction with rabbit ATG was performed.

In cases of hyperglycemia in the early postoperative period, rapid insulin therapy was initiated when the fasting capillary blood glucose level was ≥160 mg/dL. Universal prophylaxis against Pneumocystis jirovecii was provided with cotrimoxazole (80 mg trimethoprym/400 mg sufamethoxazole/day, for 3 months), and cytomegalovirus (CMV) prophylaxis was given to at-risk patients with oral valganciclovir (450 mg/12 h if eGFR > 40 mL/mn/1.73 m^2^, with the dosage adjusted appropriately for lower eGFR values). Biopsy-proven acute rejection was classified according to the Banff 2019 criteria [17] and treated with 3 boluses of 500 mg/day intravenous methylprednisolone. No corticosteroid-resistant or antibody-mediated rejection was observed during this study. Delayed graft function (DGF) was defined as the need for dialysis within the first week post-transplantation.

### 2.4. Assessment of Glucose Metabolism Alterations

Fasting blood glucose was measured at 1 week, 1 month, 2 months, and 3 months post-transplantation. A standard oral glucose tolerance test (OGTT) with 75 g glucose was performed at 3 months, except in patients who had already developed overt PTDM. Glucose and insulin levels were measured at baseline and after 30 and 120 min of glucose ingestion. Diabetes was defined according to ADA criteria [16] as fasting glucose > 125 mg/dL, the use of antidiabetic medication, or 2 h glucose ≥ 200 mg/dL. Impaired fasting glucose (IFG) was defined as fasting glucose ≥ 100 and <126 mg/dL, and impaired glucose tolerance (IGT) as 2 h glucose ≥ 140 and <200 mg/dL. IFG and IGT, isolated or combined, were considered prediabetic states (PreDM) [18]. The Insulin Sensitivity Index modified for renal transplant recipients (ISI-Tx) was calculated using the formula [6,19]: 0.208 − 0.0032 × BMI − 0.0000645 × Ins 120 − 0.00375 × Gluc 120, where Ins 120 and Gluc 120 are serum insulin (pmol/L) and plasma glucose (mmol/L) 120 min after the OGTT. The insulinogenic index (IGI) was calculated from insulin (μU/mL) and glucose (mmol/L) at 0 and 30 min and was used to estimate insulin secretory function: (Ins 30 − Ins 0)/(Gluc 30 − Gluc 0) [6,20].

### 2.5. Analytical and Other Determinations

Plasma glucose, triglycerides, HDL, LDL cholesterol, and magnesium levels were determined using a clinical chemistry analyzer (Cobas-E 801, Roche Diagnostic, Barcelona, Spain^®^). Glycated hemoglobin (HbA1c) was measured by high-performance liquid chromatography (D-100; Biorad^®^ Madrid, Spain). Insulin levels were measured using a chemiluminescence immunoassay system (Abbott Laboratories^®^, Madrid, Spain). The GFR was estimated by MDRD 4 (eGFR), and proteinuria was quantified either in 24 h urine or as the protein/creatinine ratio in the first voided morning sample by standard methods. At the end of the study, the GFR was measured by iohexol plasma clearance as previously described [21]. Whole-blood tacrolimus trough levels were determined by automated chemiluminescence assays with frequent determinations as per clinical practice (Architect Tacrolimus, Abbot, Madrid, Spain). Levels were also recorded at 1 week and at 1, 2, and 3 months.

At the end of the study, 24 h ambulatory blood pressure monitoring was performed on each group.

### 2.6. Efficacy Variables

The primary endpoint was the presence of PTDM based on ADA criteria. The incidence of PreDM (IGT and/or IFG) was assessed as a secondary outcome. Post-OGTT glucose levels were also compared between IR-Tac and LCPT groups. HbA1c was not included due to its lack of diagnostic sensitivity early after transplantation [3]. Major safety variables included biopsy-proven acute rejection rate, patient and graft survival, eGFR, and proteinuria. Treatment-emergent severe and non-severe side effects were recorded and reported to the Pharmacovigilance Unit of the Hospital Universitario de Canarias.

### 2.7. Statistical Analysis

Continuous variables are expressed as mean ± standard deviation or median plus interquartile range as appropriate; categorical variables are expressed as percentages. The Student’s *t*-test or the Mann–Whitney U test, as appropriate, was used for comparisons of two independent samples. Categorical variables were compared using the chi-square test or Fisher’s exact test. A *p*-value < 0.05 was considered statistically significant.

### 2.8. Sample Size

No comparison of IR-tacrolimus vs. LCPT with post-transplant glucose metabolic alterations as the primary endpoint has been conducted. Therefore, we planned a pilot study aimed at detecting a reduction of the composite of PTDM plus PreDM from the reported 52% three months after transplantation [2] to 20%. For a single-sided alpha of 0.05 and beta of 0.8, 27 patients per arm should be included. For this exploratory trial, we performed a per-protocol analysis.

### 2.9. Safety

Adverse events were monitored systematically and continuously. Serious adverse events were defined according to the International Conference on Harmonisation’s Guideline for Clinical Safety Data Management. Treatment-emergent severe and non-severe side effects were recorded and reported to the Pharmacovigilance Unit of the Hospital Universitario de Canarias.

Major safety variables included the biopsy-proven acute rejection rate, patient and graft survival, eGFR, and proteinuria.

## 3. Results

### 3.1. Patient Disposition

**Figure 1:**
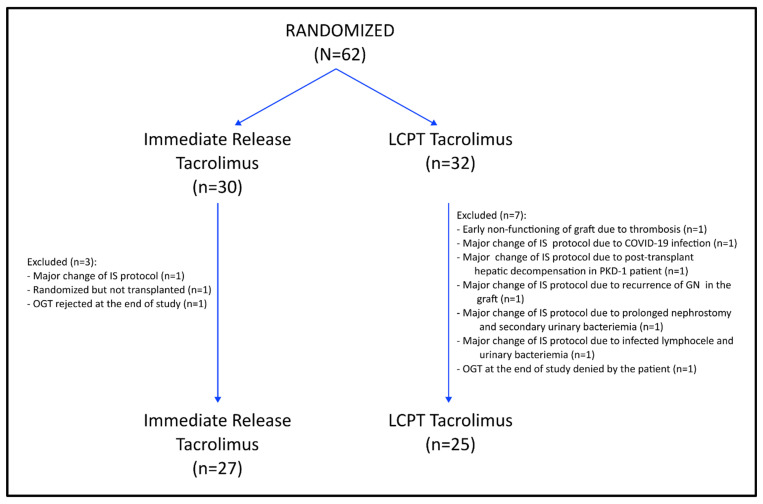
Patients’ disposition. IS: Immunosuppression; OGT: Oral glucose tolerance test. PKD-1: Autosomal Dominant Polycystic Kidney Disease type I; GN: Glomerulonephritis.

Figure 1 illustrates the patients’ disposition. A total of 62 patients were randomized, with 10 patients subsequently excluded (three from the IR-Tac group and seven from the LCPT group). The most common reason for exclusion was a violation of the immunosuppression protocol. Ultimately, 52 patients completed the study, with 27 in the IR-Tac group and 25 in the LCPT group.

### 3.2. Baseline Demographics of Recipients and Donors

Table 1 presents the baseline characteristics of recipients and donors in the two study groups. Variables associated with PTDM risk were balanced between the groups, except for BMI, which was higher in the IR-Tac group by chance. Delayed graft function was significantly more common in the IR-Tac group regardless of age, cold ischemia time, donor type (Table 1), or the use of r-ATG for induction (22.2% of patients in the IR-Tac group and 12% in the LCPT group (*p* = 0.5)).

### 3.3. Post-Transplant Evolution and Other Outcomes at the End of the Study

As shown in Table 2, three cases of biopsy-proven acute rejection were observed in the IR-Tac group, while there were none in the LCPT group. Consequently, the cumulative corticosteroid dose at the end of the study was significantly higher in the IR-Tac group. CMV and BKV infections were comparable between the groups. Due to the lower incidence of delayed graft function (DGF) in the LCPT group (Table 1), the estimated glomerular filtration rate (eGFR) at one and two months was significantly higher in the LCPT group (Table 2). Although differences at 3 months did not reach statistical significance, the mean measured GFR was 6.2 mL/min higher in the LCPT arm (Table 2). Proteinuria levels were comparable between the groups. At the end of the study, there was a trend toward lower weight gain in the IR-Tac group (Table 2). Lipid levels, blood pressure, and magnesium levels were similar between the groups. None of the included patients lost their grafts, and all survived.

### 3.4. Tacrolimus Exposure

Tacrolimus trough levels were similar throughout the study in both groups, except at 3 months when they were higher in the LCPT group (Table 3). At this time point, a higher proportion of patients in the LCPT group exhibited tacrolimus levels above the target range (7.4% vs. 40%; *p* = 0.005). Two cases of biopsy-proven acute reversible tacrolimus-related nephrotoxicity were observed in the LCPT group, while none were observed in the IR-Tac group (Table 2).

### 3.5. Primary and Secondary Efficacy Endpoints

Transient insulin therapy early after transplantation was comparable between groups: 19 out of 27 patients (70.4%) in the IR-Tac group and 13 out of 25 patients (59.4%) in the LCPT group (*p* = 0.26). Fasting glucose throughout the study was not significantly different between the groups (Table 4). Plasma glucose levels 30 min after the OGTT were significantly lower in the LCPT group (Table 4).

**Figure 2:**
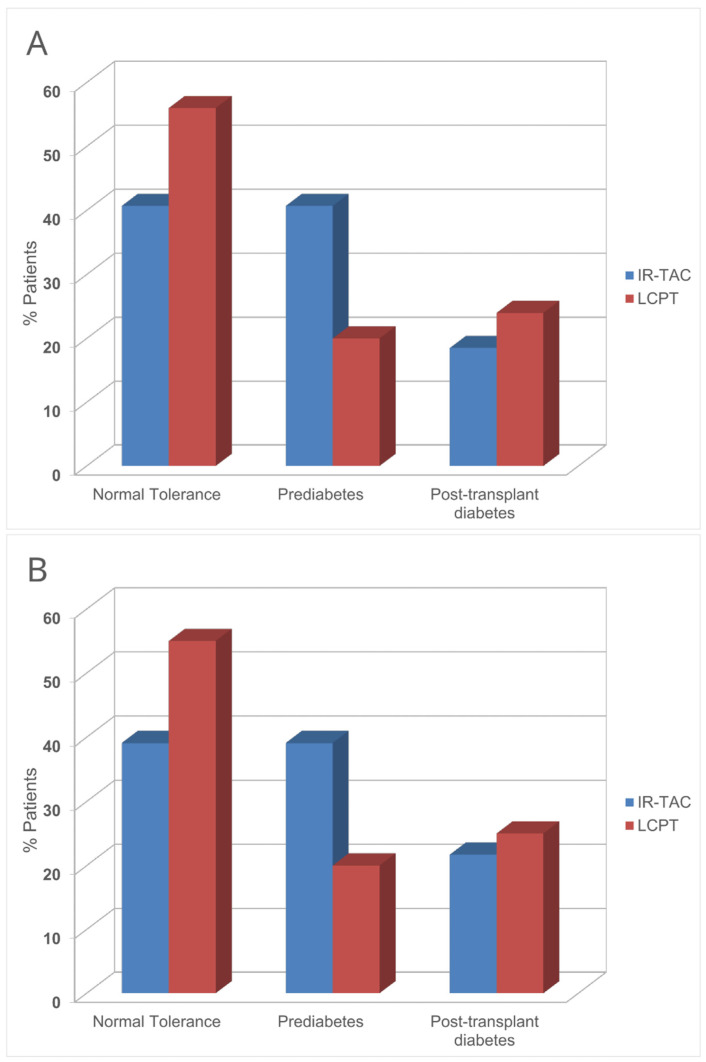
Distribution of glucose metabolism abnormalities at the end of the study in each group. Prediabetes: Impaired Fasting Glucose and Impaired Glucose Tolerance, isolated or combined. (**A**): All patients; (**B**): Excluding patients with acute rejection or a baseline BMI < 22 Kg/m^2^. IR-Tac: Immediate-release tacrolimus; LCPT: LCP Tacrolimus.

PTDM incidence was comparable between the groups (Table 4). However, normal glucose tolerance was numerically more common in the LCPT group (56% vs. 40.7%), occurring at the expense of a 50% reduction in prediabetic states (IGT and/or IFG), which did not reach statistical significance (*p* = 0.1) (Table 4; Figure 2A).

### 3.6. Sensitivity Analysis

Since all biopsy-proven acute rejections occurred in the IR-Tac group, the cumulative corticosteroid dose was higher in this group (Table 2). Additionally, by chance, baseline BMI was significantly higher in the IR-Tac group (Table 1). These differences became non-significant when excluding patients with acute rejection or the lowest values of baseline BMI (≤10th percentile: ≤22 kg/m^2^), who likely exhibit lower insulin resistance and chances of developing tacrolimus-induced PTDM or PreDM [6,8] (Supplementary Table S1).

Notably, the differences in primary and secondary outcomes between the groups remained unchanged (Supplementary Table S1; Figure 2B).

### 3.7. Adverse Events

The total number of severe and non-severe adverse events was comparable between the groups (Supplementary Table S2). In the analysis stratified by gender, urinary tract infections and overall infectious episodes were significantly lower in women in the LCPT group.

## 4. Discussion

Immediate-release tacrolimus (IR-Tac) is rapidly absorbed in the proximal small bowel, achieving peak blood concentrations 90 to 120 min after administration twice daily. This 3-month randomized pilot study investigated whether the “flatter” concentration–time curve of once-daily tacrolimus (LCPT) could mitigate beta-cell stress and glucose metabolism disturbances following renal transplantation, especially among recipients at risk for post-transplant prediabetes (PreDM) or diabetes (PTDM) [1,6,7], evaluated at the peak incidence of glucose metabolism disorders (3 months post-transplant) [2].

To our knowledge, no study has examined the clinical significance of different tacrolimus pharmacokinetics on glucose metabolism after transplantation. Thus, this pilot and exploratory study was designed with a sample size to detect a 60% reduction in combined PTDM or PreDM incidence [2]. This aimed to provide initial insight into whether LCPT might exhibit a lower incidence of glucose metabolism disorders, guiding future trials with adequate statistical power.

Plasma glucose levels following an OGTT at the study’s conclusion were numerically lower in the LCPT group, achieving statistical significance at 30 min (Table 4). Consequently, the incidence of glucose metabolism disorders at 3 months post-transplantation was reduced by 15% with LCPT, primarily due to a 50% decrease in PreDM, while no advantage was observed in PTDM incidence (Table 4; Figure 2A). This outcome may be attributed to the heightened susceptibility of patients with severely impaired beta-cell function pre-transplant to developing PTDM after exposure to tacrolimus [6], irrespective of formulation. In contrast, LCPT’s smoother pharmacokinetic profile is expected to mitigate PreDM incidence in cases of less severe beta-cell damage. The trend toward a reduction in PreDM observed in this study is noteworthy, as PreDM substantially increases long-term mortality [5], poses a cardiovascular risk almost equivalent to that of PTDM [4], and is a risk factor for the subsequent development of PTDM [2]. However, this finding has to be confirmed in future adequately powered studies. Lastly, these differences in glucose metabolism favoring LCPT were noted alongside a trend towards higher tacrolimus exposure (Table 3) and improved efficacy (lower incidence of acute rejection; Table 2).

Baseline risk factors such as a family history of diabetes, age, race, magnesium levels, or statin use [1,3,6] were balanced between groups. However, chance imbalances in pre-transplant BMI and acute rejection episodes (all occurring in the IR-Tac group) were notable confounders. Sensitivity analysis excluding patients with acute rejection or a BMI < 22 Kg/m^2^ indicated persistent between-group differences in glucose metabolism, with comparable BMI and cumulative steroid doses (Supplementary Table S1; Figure 2B), suggesting LCPT’s potential benefits independent of these factors.

The incidence of DGF was significantly lower with LCPT (Table 2). This was not attributable to differences in donor age, cold ischemia time, donor type, or induction therapy with rabbit ATG. This difference may partly stem from the timing of tacrolimus initiation. IR-Tac was started just before RT and may have contributed to ischemia-reperfusion injury, whereas LCPT was administered within 24 h post-transplantation. Additionally, the better preservation of residual renal function in PD patients, who were more prevalent in the LCPT group (Table 1), may have contributed to the reduction in DGF. As a result of the lower incidence of DGF, better renal function was observed in the LCPT group during the first two months, although this significance diminished by three months (Table 2).

Total severe and non-severe adverse events were comparable between groups (Supplementary Table S2). In a gender-stratified analysis, rates of urinary tract infections were significantly lower among women in the LCPT group. We acknowledge that without controlling for confounding variables such as the duration of bladder catheterization or ureteral stent placement, we cannot draw definitive conclusions.

This study acknowledges several limitations. Due to its exploratory nature, this study included a limited number of patients, was conducted over a relatively short period, and lacked the statistical power necessary to support recommending LCPT for the management of glucose metabolism disorders in clinical practice (beta 0.28 for an alpha of 0.05). The small sample size was likely a significant factor contributing to the unbalanced randomization of baseline BMI. The cumulative corticosteroid dose was also unevenly distributed between the groups, as all three acute rejection episodes observed in this study occurred within the IR-Tac group. Although these imbalances were addressed through a sensitivity analysis, the possibility of bias cannot be ruled out. Nonetheless, the study results indicate a promising direction, warranting further investigation into LCPT’s potential biological activity. This trial lacked a formal pharmacokinetic study to support differences in Cmax and Tmax between the two formulations. A protocol biopsy at the end of the study was not planned but could have provided morphological insights into observed graft function differences. The majority of the patients were Caucasian, so the results cannot be extrapolated to other races. The study duration was extended due to overlap with the COVID-19 pandemic, reducing transplant activity. Lastly, findings may not apply to the once-daily extended-release formulation Advagraf^®^, which differs in its pharmacokinetic profile from LCPT [12,13].

In conclusion, this exploratory trial did not succeed in demonstrating a reduction in diabetes following renal transplantation in at-risk patients treated with LCPT. Nevertheless, it does suggest that LCPT may lower the incidence of prediabetes compared to IR-Tac. The potential biological effects of LCPT in preventing glucose metabolism disorders warrant further investigation.

## Figures and Tables

**Table 1:** Baseline characteristics.

Variable	IR-TAC	LCPT	*p*-Value
	(n = 27)	(n = 25)	
Recipient Age (years)	64 (50.6–68.2)	63.52 (46.53–69.66)	0.7
Sex (% Males)	17/27 (63%)	19/25 (76%)	0.31
Race (% Caucasians)	26/27 (96.3%)	23/25 (92%)	0.58
BMI (Kg/m^2^)	28.61 ± 4.43	25.53 ± 4.19	0.013
Family history of Diabetes	7/24 (29.2%)	9/23 (39.1%)	0.47
Polycystic kidney disease	5/27 (18.5%)	6/25 (24%)	0.63
Peritoneal dialysis	7/26 (26.9%)	12/25 (48%)	0.12
Time on dialysis (months)	21.9 (15.2–32)	23.4 (9.7–28.6)	0.8
Total Cholesterol (mg/dL)	152.92 ± 38.84 (n = 26)	158.59 ± 37.27 (n = 22)	0.61
HDL cholesterol (mg/dL)	43.62 ± 13.32 (n = 21)	44.10 ± 11.42 (n = 21)	0.90
LDL cholesterol (mg/dL)	75 (56–99) (n = 19)	67 (60–98.5) (n = 21)	0.9
Triglycerides (mg/dL)	143 (102–167.8) (n = 26)	145 (103.5–181.5) (n = 22)	0.7
Tobacco (former or current smoker)	8/26 (30.8%)	13/25 (52%)	0.30
Statin treatment	19/26 (73.1%)	17/25 (68%)	0.70
Fasting glucose (mg/dL)	90.3 (83–96)	91 (83.5–94.5)	0.8
HbA1c (%)	5.19 ± 0.41 (n = 18)	5.22 ± 0.29 (n = 17)	0.85
Donor age (years)	56.70 ± 10.67	55.24 ± 13.40	0.66
Donor Sex (% Males)	17/27 (63%)	20/25 (80%)	0.18
Cold Ischemia Time (hours)	11.42 (6.54–18.67)	10.12 (7.66–16.58)	0.99
Donation after cardiac death	8/27 (29.6%)	6/25 (24%)	0.65
Delayed graft function	10/27 (37%)	2/25 (8%)	0.02

IR-TAC: Immediate-release tacrolimus; LCPT: LCP Tacrolimus.

**Table 2:** Post-Renal-Transplantation evolution and other outcomes at the end of the study.

Variable	IR-TAC	LCTP	*p*-Value
	(n = 27)	(n = 25)	
BMI (Kg/m^2^)	28.34 ± 4.64	26.02 ± 3.71	0.06
Weight increase (Kg)	−0.99 ± 4.1	0.92 ± 4.7	0.1
Total Cholesterol (mg/dL)	192.63 ± 38.52 (n = 24)	176.96 ± 40.17 (n = 23)	0.18
HDL cholesterol (mg/dL)	52.04 ± 15.24 (n = 23)	53.87 ± 16.92 (n = 23)	0.70
LDL cholesterol (mg/dL)	109.45 ± 32.16 (n = 20)	99.64 ± 33.63 (n = 22)	0.34
Triglycerides (mg/dL)	132 (101–170)	116 (100.50–143.50)	0.36
Statin therapy	9/26 (34.6%)	12/25 (48%)	0.33
Magnesium (1 week)	1.96 ± 0.3	1.9 ± 0.3	0.4
Magnesium (1 month)	1.55 ± 0.3	1.5 ± 0.15	0.7
Magnesium (2 months)	1.6 ± 0.16	1.6 ± 0.17	0.9
CNI-related acute nephrotoxicity (%)	0/27 (0%)	2/25 (8%)	0.2
Acute Rejection (%)	3/27 (11.1%)	0/25 (0%)	0.2
Cumulative corticosteroid dose (mg)	1321.25 (1189.38–1561.25)	1195 (1173.75–1270)	0.05
CMV Infection (%)	2/27 (9.4%)	2/25 (8%)	1
BKV Infection (%)	0%	1/25 (4%)	0.8
Awake SPB (ABPM) (mmHg)	129.60 ± 12.55 (n = 25)	129.84 ± 12.78 (n = 25)	0.95
Asleep SBP (ABPM) (mmHg)	124.68 ± 13.19 (n = 25)	129.48 ± 16.92 (n = 25)	0.27
Awake DBP (ABPM) (mmHg)	79.92 ± 8.11 (n = 25)	80.48 ± 8.61 (n = 25)	0.81
Asleep DBP (ABPM) (mmHg)	75.32 ± 8.46 (n = 25)	77.80 ± 8.75 (n = 25)	0.31
Creatinine (mg/dL)			
1 month	1.77 (1.48–2.48)	1.52 (1.28–1.75)	0.07
2 months	1.65 (1.43–1.95)	1.46 (1.2–1.85)	0.1
3 months	1.6 (1.36–1.86)	1.42 (1.14–1.87)	0.36
eGFR (mL/mn/1.73 m^2^)			
1 month	39.23 ± 16.57	49.16 ± 15.74	0.03
2 months	41.16 ± 14.36	48.92 ± 13.95	0.05
3 months	44.68 ± 13.99	51.28 ± 15.48	0.11
Measured GFR 3 months (mL/mn)	49.2 ± 17.3 (n = 26)	55.4 ± 18.2 (n = 23)	0.2
Proteinuria 3 months (mg/gr creatinine)	154.2 (133.2–244.2)	152.8 (139.7–271.2)	0.90

IR-TAC: Immediate-release tacrolimus; LCPT: LCP Tacrolimus. CNI: calcineurin inhibitor. CMV: Cytomegalovirus. BKV: BK virus. ABPM: 24 h ambulatory blood pressure monitoring. SBP: systolic blood pressure; DBP: diastolic blood pressure. eGFR: estimated GFR (MDRD).

**Table 3:** Tacrolimus trough levels during the study.

Whole-Blood Tacrolimus Levels (ng/mL)	IR-Tacrolimus	LCPT	*p*-Value
	(n = 27)	(n = 25)	
1 week	9.5 (7.1–12.9)	10.5 (9.4–14.5)	0.1
1 month	9.5 (7.5–11.2)	9.8 (7.6–11.7)	1
2 months	9.6 (7–12)	10.7 (8.3–13)	0.2
3 months	8.3 (6.9–9.2)	9.4 (7.4–11.4)	0.05

**Table 4:** Primary and secondary outcomes for each group.

Variables	IR-TAC	LCPT	*p*-Value
	(n = 27)	(n = 25)	
Fasting Glucose (mg/dL):			
Pre-Transplantation	90.3 (83–96)	91 (83.5–94.5)	0.77
1 week	102 (92–116)	99 (93–116)	0.88
1 month	90 (83–98)	92 (86–104)	0.69
2 months	95 (86–108)	92 (85.25–105)	0.68
3 months	92 (86–102)	88 (81–97.5)	0.24
Oral Glucose Tolerance Test (3 months)			
Baseline Fasting Glycemia (mg/dL)	92 (86–102)	88 (81–97.5)	0.24
Glycemia at 30 min (mg/dL)	157 (138–178.50) (n = 18)	139 (125–154.5) (n = 21)	0.02
Glycemia at 120 mn (mg/dL)	131 (111–167) (n = 27)	120 (97–181) (n = 23)	0.53
Baseline Insulin (mcU/mL)	7.9 (4.8–10.2) (n = 23)	7 (5.5–10) (n = 23)	0.84
Insulin at 30 min (mcU/mL)	25.35 (13.95–41.23) (n = 18)	22.9 (9–43.6) (n = 21)	0.67
Insulin at 120 min (mcU/mL)	32.35 (16.1–49) (n = 22)	27.90 (7.1–44.5) (n = 23)	0.32
Insulin Sensitivity Index (ISI)	7.4 (5.7–8.8) (n = 22)	8.5 (6.6–9.8) (n = 23)	0.2
Insulinogenic Index	43.7 (20.7–66.4) (n = 16)	38.4 (22.7–147.2) (n = 19)	0.7
DISTRIBUTION OF GLUCOSE METABOLIC ALTERATIONS AT THE END OF STUDY			
Normal Tolerance	11/27 (40.7%) (95%CI: 24.5–59%)	14/25 (56%) (95%CI: 37.1–73.3%)	0.4
Isolated impaired fasting glucose (IFG)	3/27 (11.1%) (95%CI: 3.85–28.1%)	1/25 (4%) (95%CI: 0.7–19.5%)	0.6
Impaired Glucose Tolerance (IGT)	8/27 (29.6%) (95%CI: 15.9–48.5%)	4/25 (16%) (95%CI: 6.4–34.7%)	0.3
Post-transplant diabetes	5/27 (18.5%) (95%CI: 8.2–36.7%)	6/25 (24%) (95%CI: 11.5–43.4%)	0.7
Prediabetes (IFG + IGT)	11/27 (40.7%) (95%CI: 25–59%)	5/25 (20%) (95%CI: 9–39%)	0.1

## Data Availability

The data presented in this study are available on request from the corresponding author.

## Supplementary Materials

The following supporting information can be downloaded at: https://www.mdpi.com/article/10.3390/jcm13247802/s1, Table S1: Sensitivity analysis; Table S2: Adverse events.
