# Supplementary material for: Efficacy of the Once-Daily Tacrolimus Formulation LCPT Compared to the Immediate-Release Formulation in Preventing Early Post-Transplant Diabetes in High-Risk Kidney Transplant Patients: A Randomized, Controlled, Open-Label Pilot Study (EUDRACT: 2017-000718-52)
Source: J Clin Med. 2024 Dec 20;13(24):7802. doi: 10.3390/jcm13247802 (PMC11728219; doi:10.3390/jcm13247802)
Supplement: Supplementary file 1 [file jcm-13-07802-s001.zip › jcm-3339341-supplementary.pdf]

Supplementary material for article:

EFFICACY OF ONCE-DAILY TACROLIMUS FORMULATION LCPT IN PREVENTING EARLY POST-TRANSPLANT DIABETES IN HIGH-RISK KIDNEY TRANSPLANT PATIENTS: A RANDOMIZED, CONTROLLED, OPEN-LABEL PILOT STUDY (EUDRACT: 2017-000718-52)

AUTHORS:

Armando Torres[1,2], Concepción Rodríguez-Adanero[1] , Constantino Fernández-Rivera[3], Domingo Marrero-Miranda[1], Eduardo de Bonis-Redondo[1], Aurelio P Rodríguez-Hernández[1], Lourdes Pérez-Tamajón[1], Ana González-Rinne[1], Diego Álvarez-Sosa[1], Alejandra Álvarez-González[1], Nuria Sanchez-Dorta[1], Estefanía Pérez-Carreño[4], Laura Díaz-Martín[4], Sergio Luis-Lima[5], Ana E. Rodríguez-Rodríguez[2], Antonia María de Vera González[5], Cristina Romero-Delgado[6], María Calvo-Rodríguez[3], Rocío Seijo-Bestilleiro[3], Consuelo Rodríguez-Jiménez[7], Domingo Hernández-Marrero[1,2], Esteban Porrini[2].

AFFILIATION:

1. Nephrology Service. Hospital Universitario de Canarias, La Laguna, Spain. atorresram@gmail.com (AT); c.rguez.adanero@gmail.com (CRA); dmarrero72@hotmail.com (DMM); ebonis@telefonica.net (EBR); aureliopas@hotmail.com (APRH); mpertam28@gmail.com (LPT); rinneanag@yahoo.es (AGR); diegoalvarezsosa@yahoo.es (DAS); alejandramag73@gmail.com (AAG); nuriasanchezdorta@gmail.com (NSD); domingohernandez@gmail.com (DHM).
2. Instituto de Tecnologías Biomédicas (ITB)-Universidad de La Laguna, La Laguna, Spain. atorresram@gmail.com (AT); domingohernandez@gmail.com (DHM); estebanporrini72@hotmail.com (EP); anarrguez@gmail.com (AERR).
3. Complejo Hospitalario Universitario de A Coruña. La Coruña, Spain. constantino.fernandez.rivera@sergas.es (CFR). maria.calvo.rodriguez@sergas.es (MCR). rocio.seijo.bestilleiro@sergas.es (RSB)
4. Research Unit. Hospital Universitario de Canarias, La Laguna, Spain. lauradiazmart@gmail.com (LDM); estefaniaperezc@gmail.com (EPC).
5. Central Laboratory. Hospital Universitario de Canarias, La Laguna, Spain. luis.lima.sergio@gmail.com (SLL); adeverag@gmail.com (AMV)
6. Pharmacy Service. Hospital Universitario de Canarias, La Laguna, Spain. cromdels@gobiernodecanarias.org (CRD)
7. Pharmacology Service. Hospital Universitario de Canarias, La Laguna, Spain. conrodjim@gmail.com (CRJ).

Corresponding Author: Armando Torres; atorresram@gmail.com

Content: Two tables

Supplementary Table 1 on page 3

Sensitivity analysis: comparison of IR-Tac vs LCPT formulations after excluding patients with a biopsy-proven acute rejection and those with a BMI<22 Kg/m2 at baseline.

Supplementary Table 2 on page 5

Safety data

SUPPLEMENTARY TABLE S1. Sensitivity analysis: comparison of IR-Tac vs LCPT formulations after excluding patients with a biopsy-proven acute rejection and those with a BMI<22 Kg/m2 at baseline.

	**IR-TAC (n=23)**	**LCPT (n=20)**	**P-VALUE**
**Recipient´s age (years)**	65.63 (50.6-68.2)	62.03 (42-68.7)	0.34
**Sex (%Males)**	16/23 (69.6%)	16/20 (80%)	0.5
**Baseline BMI (Kg/m^2)**	28.5 ± 3.6	26.8 ± 3.6	0.14
**Pretransplant Fasting Glucose (mg/dl)**	88 (83-96)	91 (86-96)	0.38
**Pretransplant HbA1c (%)**	5.2±0.4	5.2±0.3	0.97
**Pretransplant total Cholesterol (mg/dl)**	146.5±37.2	157.6±37.6	0.36
**Pretransplant Triglycerides (mg/dl)**	103.5 (101.8-160.5)	154.5 (140.8-192.5)	0.06
**Pretransplant HDL-cholesterol (mg/dl)**	41.6±11.4	42.2±1.9	0.87
**PreTransplant LDL-cholesterol (mg/dl)**	75 (53-99)	71 (59.3-104.3)	0.7
**Time on Dialysis (months)**	22.3 (17.4-31)	23.7 (9.6-27.2)	0.38
**Donor age (years)**	56.4±10.1	53.5±12.7	0.4
**Cold Ischemia Time (hours)**	11.4 (6.5-19.6)	11.05 (7.5-16.95)	0.96
**Delayed graft function (%)**	9/23 (39.1%)	2/20 (10%)	0.039
**Cumulative corticosteroids dose (mg) 3 months**	1287.5 (1180-1422.5)	1195 (1178-1253.8)	0.12
**Tacrolimus (ng/ml)**			
**1 week**	10.1 (7.7-13.1)	10.45 (9.3-13.2)	0.5
**1 month**	9.9 (7.5-11.2)	9.95 (7.3-12.03)	0.9
**2 months**	9.6 (7-11.4)	10.8 (7.9-12.9)	0.18
**3 months**	8.5 (7.2-9.6)	9.2 (7.4-11.4)	0.2
**Magnesium 1 week**	1.98±0.3	1.88±0.3	0.3
**Magnesium 1month**	1.6±0.2	1.5±0.2	0.6
**Magnesium 2 months**	1.6±0.1	1.6±0.2	0.8
**Statin Therapy**	9/23 (39.1%)	10/20(50%)	0.5
**eGFR (ml/mn/1.73m2)**			
**1 month**	39.2±16.1	47.8±16	0.09
**2 months**	43.9±13.4	46.8±13.8	0.49
**3 months**	46.2±14.5	48.3±14.4	0.6
**Measured GFR (3 months)**	51.3±17.5	55.1±19.1	0.5
**Proteinuria 3 months**	162 (136.7-242.8)	157.8 (142.4-305.7)	0.69
**Primary and 2ry outcomes**			
**Normal Tolerance**	9/23 (39.1%) (95%CI: 22.2-59.2%)	11/20 (55%) (95%CI: 34.2-74.2%)	0.37
**Isolated Impaired Fasting Glucose (IFG) (%)**	2/23 (8.7%) (95%CI: 2.4-26.8%)	1/20 (5%) (95%CI: 0.9-23.6%)	1
**Impaired Glucose Tolerance (IGT) (%)**	7/23 (30.4%) (95%CI: 15.6-51%)	3/20 (15%) (95%CI: 5.2-36%)	0.29

**Post-transplant Diabetes (%)**	5/23 (21.7%) (95%CI: 9.7-41.9%)	5/20 (25%) (95%CI: 11.2-46.9%)	1
**PreDiabetes (IFG+IGT)**	9/23 (39.1%)	4/20 (20%)	0.2
**Post-Transplant Fasting Glucose (mg/dl)**			
**1 week**	99 (92-116)	99 (92.5-110.8)	0.9
**1 month**	90 (87-98)	94 (86.3-107)	0.7
**2 months**	95 (86-106)	91 (85.3-102.8)	0.7
**3 months**	92 (85-100)	87 (80.5-101.8)	0.4
**Oral Glucose Tolerance Test (3 months)**			
**Glycemia t0**	92 (85-100)	87 (80.5-101.8)	0.4
**Glycemia t30**	159 (138-176)	140 (129-155.8)	0.03
**Glycemia t120**	132 (111-167)	122.5 (93-175.5)	0.39
**Insulin t0**	8 (5.4-10.2)	8.6 (5.4-11.4)	0.78
**Insulin t30**	26 (14.1-42.2)	22.6 (7.4-47.2)	0.65
**Insulin t120**	40 (20.2-55.4)	27.9 (2.7-43.1)	0.12
**Insulin Sensitivity Index**	6.9 (4.8-8.3)	8.3 (6.3-9.5)	0.2
**Insulinogenic Index**	46.7 (22.4-64)	48.4 (21.9-176.4)	0.7

t0: baseline; t30 and t120: 30 and 120 mn after oral glucose tolerance test.

SUPPLEMENTARY TABLE S2. Safety data.

	IR-TAC		LCPT		ALL		P value
	Affected/ Exposed (%)	Events	Affected/ Exposed (%)	Events	Affected/ Exposed (%)	Events	
**INFECTIONS**							
UTI without bacteriemia							
All	11/27 (40.7%)	11	5/25 (20%)	5	16/52 (30.8%)	16	0,1
Women	6/10 (60%)	6	1/6 (16.7%)	1	7/16 (43.8%)	7	0.2
Men	5/17 (29.4%)	5	4/19 (21.1%)	4	9/36 (25%)	9	0.7
UTI with bacteriemia							
All	2/27 (7.4%)	3	0/25 (0%)	0	2/52 (3.8%)	3	0.2
Women	2/10 (20%)	3	0/6 (0%)	0	2/16 (12.4%)	3	0.6
Men	0/17 (0%)	0	0/19 (0%)	0	0/36 (0%)	0	1
Total UTI							
All	13/27 (48.1%)	14	5/25 (20%)	5	18/52 (34.6%)	19	0.03
Women	8/10 (80%)	9	1/6 (16.7%)	1	9/16 (56.2%)	10	0.03
Men	5/17 (29.4%)	5	4/19 (21.1%)	4	9/36 (25%)	9	0.7
Bacteriemia (all cause)							
All	4/27 (14.8%)	5	0/25 (0%)	0	4/52 (7.7%)	5	0.047
Women	3/10 (30%)	4	0/6 (0%)	0	3/16 (18.7%)	4	0.4
Men	1/17 (5.9%)	1	0/19 (0%)	0	1/36 (2.8%)	1	0.8
Pneumonia							
All	2/27 (7.4%)	2	0/25 (0%)	0	2/52 (3.8%)	2	0.49
Women	1/10 (10%)	1	0/6 (0%)	0	1/16 (6.2%)	1	1
Men	1/17 (5.9%)	1	0/19 (0%)	0	1/36 (2.8%)	1	0.5
CMV							
All	2/27 (7.4%)	2	2/25 (8%)	2	4/52 (7.7%)	4	1
Women	0/10 (0%)	0	1/6 (16.7%)	1	1/16 (6.2%)	1	0.4
Men	2/17 (11.8%)	2	1/19 (5.3%)	1	3/36 (8.3%)	3	0.6
BKV							
All	0/27 (0%)	0	1/25 (4%)	1	1/52 (1.9%)	1	0.8
Women	0/10 (0%)	0	0/6 (0%)	0	0/16 (0%)	0	1
Men	0/17 (0%)	0	1/19 (5.3%)	1	1/36 (2.8%)	1	1
Other Infections							
All	3/27 (11.1%)	3	2/25 (8%)	2	5/52(9.6%)	5	1
Women	1/10 (10%)	1	0/6 (0%)	0	1/16 (6.2%)	1	1
Men	2/17 (11.8%)	2	2/19 (10.5%)	2	4/36 (11.1%)	4	1
Total Infections							
All	19/27 (70.3%)	24	10/25 (40%)	10	29/52 (55.8%)	34	0.01
Women	10/10 (100%)	12	2/6 (33.3%)	2	12/16 (75%)	14	0.02
Men	9/17 (53%)	12	8/19 (42.1%)	8	17/36 (47.2%)	20	0.4
**GRAFT RELATED**							
ACUTE REJECTION							

All	3/27 (11.1%)	3	0/25 (0%)	0	3/52 (5.8%)	3	0.2
Women	2/10 (20%)	2	0/6 (0%)	0	2/16 (12.5%)	2	0.5
Men	1/17 (5.9%)	1	0/19 (0%)	0	1/36 (2.8%)	1	0.5
NEFROTOXI-CITY							
All	0/27 (0%)	0	2/25 (8%)	2	2/52 (3.8%)	2	0.2
Women	0/10 (10%)	0	0/6 (0%)	0	0/16 (6.2%)	0	1
Men	0/17 (0%)	0	2/19 (10.5%)	2	2/36 (5.6%)	2	0.5
GRAFT BIOPSY BY INDICATION							
All	8/27 (28.6%)	8	6/25 (25%)	6	14/52 (26.9%)	14	0.8
Women	4/10 (40%)	4	2/6 (33.3%)	2	6/16 (37.5%)	6	0.9
Men	4/17 (23.5%)	4	4/19 (21.1%)	4	2/36 (22.2%)	8	1
WOUND COLECTIONS							
All	7/27 (25.9%)	7	0/25 (0%)	0	7/52 (13.5%)	7	0.01
Women	1/10 (10%)	1	0/6 (0%)	0	1/16 (6.2%)	1	1
Men	6/17 (35.3%)	6	0/19 (0%)	0	6/36 (16.7%)	6	0.01
OBSTRUCTIVE UROPATHY							
All	0/27 (0%)	0	2/25 (8%)	2	2/52 (3.8%)	2	0.2
Women	0/10 (0%)	0	1/6 (16.7%)	1	1/16 (6.2%)	1	0.4
Men	0/17 (0%)	0	1/19 (5.3%)	1	1/36 (2.8%)	1	1
**NEUTRO-PENIA**							
All	4/27 (14.8%)	4	5/25 (20%)	5	9/52 (17.3%)	9	0.7
Women	1/10 (10%)	1	1/6 (16.7%)	1	2/16 (12.5%)	2	1
Men	3/17 (17.6%)	3	4/19 (21.1%)	4	7/36 (19.4%)	7	0.9
**CARDIO-VASCULAR**							
All	1/27 (3.7%)	1	1/25 (4%)	2	2/52 (3.8%)	3	0.4
Women	0/10 (0%)	0	0/6 (0%)	0	0/16 (0%)	0	1
Men	1/17 (5.9%)	1	1/19 (5.3%)	2	2/36 (5.6%)	3	0.4
**OTHER**							
All	5/27 (18.5%)	5	4/25 (16%)	4	9/52 (17.3%)	9	0.6
Women	3/10 (30%)	3	0/6 (0%)	0	3/16 (18.8%)	3	0.3
Men	2/17 (11.8%)	2	4/19 (21.1%)	4	6/36 (16.7%)	6	0.7
**SEVERE ADVERSE EVENTS**							
All	16/27 (59.3%)	36	12/25 (48%)	19	28/52 (53.8%)	55	0.2
Women	7/10 (70%)	16	2/6 (33.3%)	2	9/16 (56.2%)	18	0,1
Men	9/17 (52.9%)	20	10/19 (52.6%)	17	19/36 (52.8%)	37	0.8
**NON-SEVERE ADVERSE ADVENTS**							
All	12/27 (44.4%)	12	8/25 (32%)	9	20/52 (38.5%)	21	0.4
Women	5/10 (50%)	5	3/6 (50%)	3	8/16 (50%)	8	1
Men	7/17 (41.1%)	7	5/19 (26.3%)	6	12/36 (33.3%)	13	0.5

UTI: Urinary tract infection. CMV: Cytomegalovirus. BKV: BK virus.
